# Crosstalk Between Iron and Sulfur Homeostasis Networks in *Arabidopsis*

**DOI:** 10.3389/fpls.2022.878418

**Published:** 2022-06-09

**Authors:** Muhammad Sayyar Khan, Qiao Lu, Man Cui, Hala Rajab, Huilan Wu, Tuanyao Chai, Hong-Qing Ling

**Affiliations:** ^1^The State Key Laboratory of Plant Cell and Chromosome Engineering, National Center for Plant Gene Research (Beijing), Institute of Genetics and Developmental Biology, Chinese Academy of Sciences, Beijing, China; ^2^Institute of Biotechnology and Genetic Engineering, The University of Agriculture Peshawar, Peshawar, Pakistan; ^3^College of Life Sciences, University of Chinese Academy of Sciences, Beijing, China

**Keywords:** iron, sulfur, crosstalk, homeostasis, *Arabidopsis*

## Abstract

The widespread deficiency of iron (Fe) and sulfur (S) is becoming a global concern. The underlying mechanisms regulating Fe and S sensing and signaling have not been well understood. We investigated the crosstalk between Fe and S using mutants impaired in Fe homeostasis, sulfate assimilation, and glutathione (GSH) biosynthesis. We showed that chlorosis symptoms induced by Fe deficiency were not directly related to the endogenous GSH levels. We found dynamic crosstalk between Fe and S networks and more interestingly observed that the upregulated expression of *IRT1* and *FRO2* under S deficiency in Col-0 was missing in the *cad2-1* mutant background, which suggests that under S deficiency, the expression of *IRT1* and *FRO2* was directly or indirectly dependent on GSH. Interestingly, the bottleneck in sulfite reduction led to a constitutively higher *IRT1* expression in the *sir1-1* mutant. While the high-affinity sulfate transporter (*Sultr1;2*) was upregulated under Fe deficiency in the roots, the low-affinity sulfate transporters (*Sultr2;1,* and *Sultr2;2*) were down-regulated in the shoots of Col-0 seedlings. Moreover, the expression analysis of some of the key players in the Fe–S cluster assembly revealed that the expression of the so-called Fe donor in mitochondria (*AtFH*) and S mobilizer of group II cysteine desulfurase in plastids (*AtNFS2*) were upregulated under Fe deficiency in Col-0. Our qPCR data and ChIP-qPCR experiments suggested that the expression of *AtFH* is likely under the transcriptional regulation of the central transcription factor *FIT.*

## Introduction

Iron (Fe) and sulfur (S) are essential nutrients for plants. Plant responses to the limited supply of S or Fe have been extensively investigated with different experimental approaches (Lewandowska and Sirko, 2008; Watanabe et al., 2010). Since both of them are closely linked, the deficiency of one nutrient is supposed to regulate the uptake and availability of the other (Forieri et al., 2013; Zuchi et al., 2015). Therefore, the crosstalk between Fe and S has sparked interest. S deficiency has become a major concern in plant nutrition in recent years, especially due to decreased emission of SO_2_ and lower S supply through mineral fertilization (McGrath et al., 2003). The responses of the plants to the S deficiency have been well documented in numerous studies (Hirai et al., 2003; Nikiforova et al., 2005; Watanabe et al., 2010). However, the mechanisms governing Fe and S sensing/signaling are far from being well understood. In contrast to soils that are depleted in S, Fe is abundant in soil, but its availability is very poor due to its insolubility in the soil matrix. Most of the studies aiming to elucidate Fe or S deficiency responses in plants were mainly focused on a single nutrient deficiency in *Arabidopsis* with few exceptions (Zuchi et al., 2009, 2015; Pii et al., 2015). However, in an agroecosystem, plants are likely subjected to the simultaneous deficiency of both nutrients, and it is reasonable to assume that the simultaneous limitation of the two nutrients may trigger responses quite distinct from those triggered by the individual limitations of the two nutrients. For example, earlier reports have shown that Fe deficiency induced more ethylene production in tomato roots, but this did not take place under dual starvation of Fe and S (Zuchi et al., 2009), suggesting that S deficiency alters the typical Fe deficiency responses in tomato (Zuchi et al., 2015). Other reports have demonstrated that the availability of S influences Fe uptake and that Fe deficiency results in modulation of S uptake and assimilation (Zuchi et al., 2009, 2015; Forieri et al., 2013). Since most of the metabolically active Fe is mainly conjugated with S to form Fe–S clusters, the provision of substrates (i.e., chelated Fe and reduced S in the form of cysteine) must be tightly regulated to meet the changing demands of plants for the assembly of Fe–S clusters and to avoid potentially toxic free Fe and sulfide (Forieri et al., 2013).

Recently, the correlation of an essential S-containing compound named glutathione (GSH) with tolerance to Fe has been demonstrated in *Arabidopsis* (Shanmugam et al., 2015). The GSH-deficient mutant *zir1* showed sensitivity to Fe limitation, whereas overexpression of *GSH* increased tolerance to Fe deficiency. These findings suggest that GSH plays an essential role in Fe-limited conditions. Moreover, this study further demonstrated that GSH-deficient mutants accumulated lower levels of Fe under Fe-limited conditions compared to wild-type plants. Interestingly, significantly higher Fe translocation from roots to shoots has been reported in GSH-overproduced *Brassica napus* lines. This study implied that GSH overproduction *via* the overexpression of the key primary S metabolism-related genes may be a biotechnological means to increase Fe in plants (Rajab et al., 2020).

There is accumulating evidence to support that Fe and S do interact with each other and that the deficiency of one nutrient may affect the uptake and availability of the other. Moreover, plants exposed to the simultaneous deficiency of S and Fe in different agroecosystems may trigger more complex responses. It is, therefore, imperative not only to understand the impact of deficiency of one nutrient over the other but also to understand how plants react to dual Fe and S deficiency. The present work aimed to study the crosstalk between Fe and S in terms of individual and dual nutrient deficiencies *via* various molecular and experimental approaches using *Arabidopsis* mutants impaired in Fe homeostasis, sulfite reduction, and GSH biosynthesis.

## Materials and Methods

### Plant Material

The seeds of *Arabidopsis* mutant impaired in Fe homeostasis (*fit1-2*), sulfite reduction (*sir1-1*), and GSH biosynthesis (*cad2-1,* and *sir1-1X cad2-1*) were surface sterilized with 10% commercial bleach solution containing 0.1% Triton-X 100 for 15 min and then washed with sterile water 3–4 times. After 3 days of cold stratification at 4°C, the seeds were plated on solidified half-strength Murashige and Skoog (MS) medium plates consisting of 2.165 g/l MS basal salt mixture (PhytoTech United States), 0.5 g/l MES, 2% sucrose, 1% agar, pH 5.8. The seedlings were grown at 22°C under long-day conditions (16 h light and 8 h dark) for 7 days. 7-day-old seedlings were then used for further analysis.

### Treatments of Fe and S Deficiency Stress

One-week old seedlings were transferred to half-strength MS medium with Fe and S (1/2MS + Fe + S), without Fe supply (1/2MS-Fe + S), without S supply (1/2MS + Fe-S), and without Fe and S supply (1/2MS-Fe-S) and cultivated for 4 days at 22°C under long-day conditions (16 h light and 8 h dark). The performance of the wild type (Col-0) and mutant lines under deficient conditions of nutrients were judged by documenting their phenotypes with the help of a digital camera.

### Chlorophyll Measurement

One-week-old seedlings of Col-0, *fit1-2*, *sir1-1*, *cad2-1*, and *s1c2* grown on 1/2 MS were transferred to treatment plates for another 4 days. All shoots were collected, and fresh weights were determined. Chlorophyll was extracted in 10 ml of 90% acetone in the dark at room temperature. The supernatant was subjected to spectrophotometry at 647 and 664 nm. The total chlorophyll content was calculated as described previously (Jeffrey and Humphrey, 1975).

### Root Length Measurement

One-week-old seedlings of Col-0, *fit1-2*, *sir1-1*, *cad2-1*, and *s1c2* grown on 1/2 MS were transferred to treatment plates for another 4 days and photographs were taken. Root length of seedlings was measured by Image J. For each experiment, at least 10 seedlings were measured for each repeat. Mean values of the three independent experiments were given.

### RNA Extraction and Quantitative Real-Time PCR

Total RNA was extracted using TRIzol Reagent (Invitrogen, United States) and treated with DNase I (Ambion, United States) to eliminate genomic DNA contamination. Revert Aid First Strand cDNA Synthesis kit (Thermo Scientific, United States) was used to synthesize first-strand cDNA from total RNA. Gene expression analysis was performed on the roots and shoots of the seedlings exposed to individual and dual Fe and S stress for 4 days. Quantitative real-time PCR was performed on a LightCycler480 machine (Roche Diagnostics, Switzerland) with the gene-specific primers (Supplementary Table S1) and SYBR Premix Ex Taq polymerase (Takara, Japan). The relative expression levels of genes were calculated by the 2^−Δ(ΔCt)^ method using AtGADPH expression as a standard.

### Chromatin Immunoprecipitation Assay

For quantitative Chromatin Immunoprecipitation (ChIP)-PCR assays, the roots of 35S:FIT-GFP (expressing the fusion protein FIT-GFP in wild type Col-0) were harvested after growing on half-strength MS agar plates without iron supply for 7 d. ChIP was performed as described by Bowler et al. (2004). The antibody against GFP was used to immunoprecipitate DNA/protein complexes from the chromatin preparation. DNA precipitated from the complexes was recovered, purified, and analyzed using the multiple-quantitative ChIP-PCR method, as described by Fan et al. (2014). The primers were designed to amplify fragments of 140–390 bp within the promoter region of genes and are provided in Supplementary Table S1.

### Statistical Analysis

The statistics for the experimental data was performed using GraphPad Prism 8.0.1. The data were analyzed by two Way Repeated Measures Analysis of Variance (Two-Way ANOVA) followed by the Dunnett’s test for the comparisons of all groups with the Col-0 control group.

## Results

### Effect of Fe and S Deficiency Stress on the Phenotype of Seedlings

**Figure 1:**
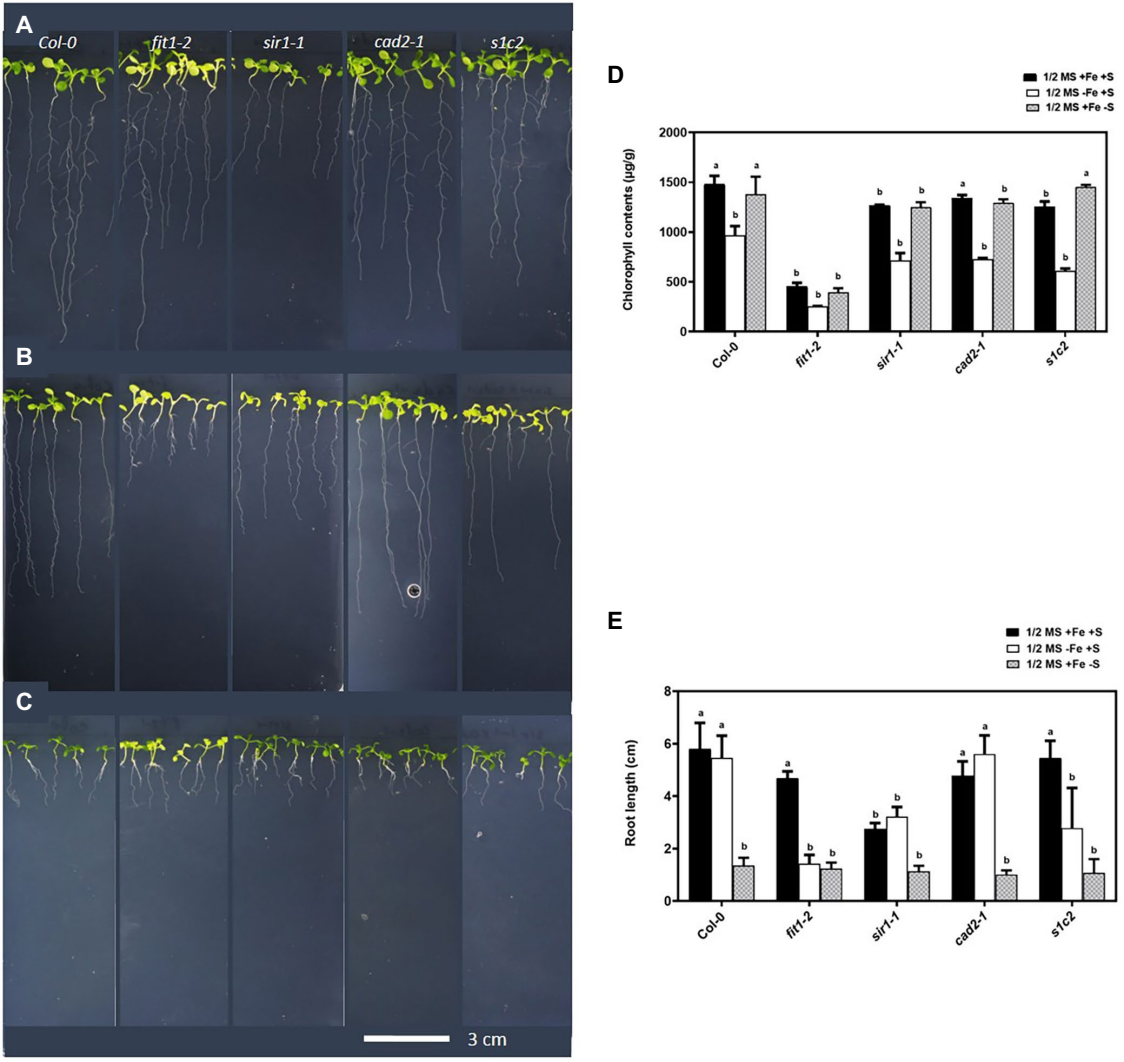
Phenotype of *Arabidopsis* seedlings exposed to Fe and S starvation. Top view of the 1-week old seedlings transferred to: **(A)** half-strength MS medium (1/2 MS), **(B)** 1/2 MS − Fe, **(C)** 1/2 MS − S for 4 days under long-day conditions. **(D)** Chlorophyll contents, **(E)** root length. Letters indicate the statistically significant differences between the wild type (Col-0) on ½ MS + Fe + S with the other treatments in Col-0 and mutants, determined with the two-way ANOVA test followed by Dunnett’s test (*p* < 0.05, *n* = 3).

The physiological responses of GSH-deficient mutants *zir1*, *cad2-1*, and *pad2-1* under Fe-deficient conditions have been previously reported (Shanmugam et al., 2015). These findings suggested that *zir1*, *cad2-1*, and *pad2-1* mutants were more sensitive to Fe deficiency, and the endogenous levels of GSH in these mutants were associated with the Fe-sensitive phenotypes under Fe deficiency. To further investigate the association of steady-state levels of GSH with sensitivity to Fe limitation and its possible crosstalk with S metabolism, we used Fe-homeostasis related mutant *fit1-2* and mutant impaired in sulfite reduction *sir1-1* (Khan et al., 2010) along with *cad2-1* (Cobbett et al., 1998), and the double mutant *sir1-1Xcad2-1* (Speiser et al., 2018) in our study. The double mutant *sir1-1Xcad2-1* will be hereafter referred to as *s1c2*. It should be noted that despite severe sulfite reduction, the steady-state levels of GSH in the *sir1-1* mutants were still similar to Col-0 (Khan et al., 2010). After 4 days of exposure to Fe-deficient conditions, *sir1-1* and *s1c2* showed more chlorosis compared with Col-0 (Figures 1B,D). The *cad2-1* mutant, which has 39% of the wild-type level of GSH, also looked paler than that of Col-0, but the extent of chlorosis was lower compared to *s1c2.* Similarly, exposure of the seedlings to S-deficient conditions for 4 days led to a significant reduction in the overall growth of all the lines, including Col-0 (Figures 1C,E). However, the seedlings of all the mutants including Col-0 looked greener, compared to the seedlings growing on the Fe-deficient media.

Direct germination of seeds on Fe-deficient media for 2 weeks revealed an obvious Fe-sensitive phenotype for *sir1-1* and *s1c2* compared to Col-0 (Supplementary Figure S1). The *cad2-1* mutant did not exhibit such Fe-sensitive phenotype. The *s1c2* mutant was more chlorotic compared to *sir1-1*. The chlorosis became more and more pronounced over time under Fe-deficient conditions.

### Expression Analysis of the Fe-Homeostasis Related Genes in the Roots of *Arabidopsis*

**Figure 2:**
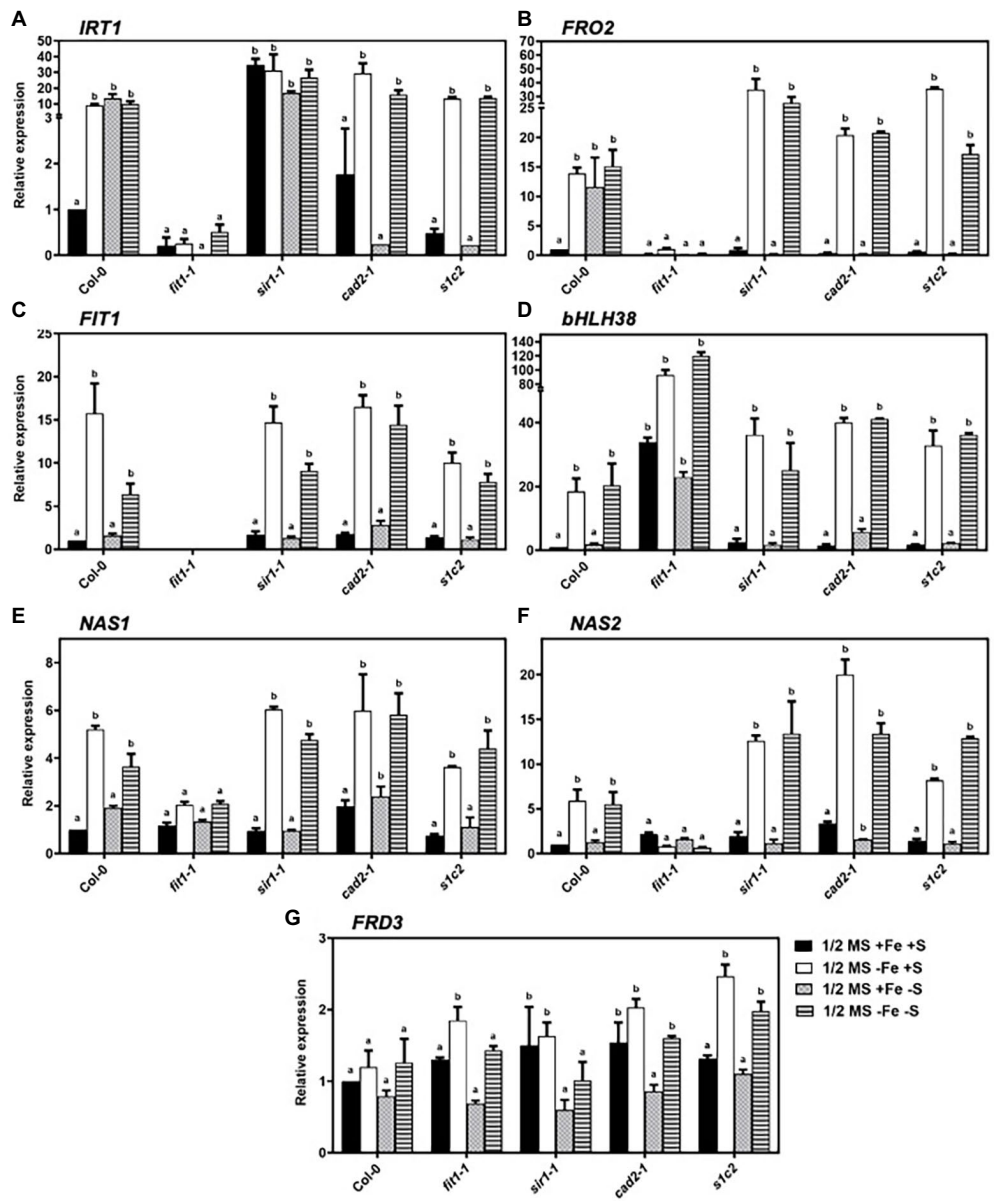
Expression profiles of the genes related to iron-homeostasis in *Arabidopsis* roots. Relative expression by quantitative RT-PCR of *IRT1* **(A)**, *FRO2* **(B)**, *FIT* **(C)**, *bHLH38* **(D)**, *NAS1* **(E)**, *NAS2* **(F)**, and *FRD3* **(G)** in the roots of *Arabidopsis* wild type (Col-0), *fit1-2*, *sir1-1*, *cad2-1*, and *s1c2* lines of 1-week old seedlings exposed to half-strength MS medium (1/2 MS + Fe + S), Fe-deficient (1/2 MS − Fe + S), S-deficient (1/2 MS + Fe − S), and Fe and S-deficient (1/2 MS − Fe − S) media for 4 days under long-day conditions. The *y*-axis shows RNA levels normalized to that of *GADPH*. **(A–G)** Letters indicate the statistically significant differences between the wild type (Col-0) on ½ MS + Fe + S with the other treatments in Col-0 and mutants, determined with the two-way ANOVA test followed by Dunnett’s test (*p* < 0.05, *n* = 3). Bars represent means ± SD.

In strategy-I plants like *Arabidopsis,* the Fe-regulated metal transporter (*IRT1*) and ferric chelate reductase (*FRO2*) are induced in the root epidermal cells in response to Fe deficiency (Connolly and Guerinot, 2002; Connolly et al., 2003; Schmidt, 2003), partly under the transcriptional regulation of the basic helix–loop–helix transcription factor FER-LIKE IRON DEFICIENCY-INDUCED FACTOR (FIT; Bauer et al., 2007). FIT is a central transcription factor and is itself upregulated by Fe deficiency (Colangelo and Guerinot, 2004; Yuan et al., 2005). In addition to Fe limitation, we analyzed the expression of the genes related to Fe uptake under dual (Fe and S) and individual (S) nutrient limitations. Apart from Fe deficiency, S deficiency also upregulated the expression of *IRT1* in Col-0 (Figure 2A). Quite remarkably, the limitation of sulfate assimilation due to the lack of sulfite reduction in *sir1-1* mutant (Khan et al., 2010) constantly activated *IRT1* expression. Interestingly, the upregulated expression of *IRT1* under S deficiency was directly or indirectly dependent on GSH because the upregulated expression of *IRT1* disappeared in the *cad2-1* mutant.

Just like *IRT1*, the expression of *FRO2* was induced under S deficiency in addition to Fe limitation in Col-0 (Figure 2B). However, unlike *IRT1*, the lack of sulfite reduction in *sir1-1* mutant did not constantly activate *FRO2* expression. Moreover, the disappearance of upregulated *FRO2* expression under S deficiency in *sir1-1*, *cad2-1,* and *s1c2* suggested that the expression of *FRO2* was also directly or indirectly dependent on *GSH1* and/or *SIR.* Under sole Fe and combined Fe and S deficiency, the expression of *FIT* and one of its activating partners called basic helix–loop–helix protein *bHLH38* was induced in all lines (Figures 2C,D). However, unlike *IRT1* and *FRO2*, the expression of *FIT* and *bHLH38* did not change under S deficiency in Col-0. Under the dual deficiency of Fe and S, the expression levels of *IRT1*, *FRO2*, *FIT*, and *bHLH38* were statistically similar to those observed under Fe deficiency.

As a major chelator of ferrous ions, nicotianamine (NA) plays a critical role in long-distance transport of Fe and transfer within the cell. The induction of two *nicotianamine synthetase* genes, *NAS1*, and *NAS2*, under Fe-starved conditions in *Arabidopsis* has already been reported (Kim et al., 2005; Klatte et al., 2009). In addition to Fe deficiency, we analyzed the expression of *NAS1* and *NAS2* in response to S deficiency and dual Fe and S deficiency. The expression of *NAS1* was induced by Fe deficiency in wild type and all mutants (Figure 2E). In response to S deficiency, the expression of *NAS1* was 1.9-fold higher in Col-0 compared to control conditions. However, in the mutant backgrounds, S deficiency did not cause any differential regulations of *NAS1* except *cad2-1*. Moreover, compared to individual Fe deficiency, dual starvation of Fe and S did not have any overriding effect on the expression of *NAS1* gene in the roots. Under normal growth conditions, the expression of *NAS2* gene was 2.1-fold, 1.9-fold, and 3.3-fold higher in *fit1-2*, *sir1-1*, and *cad2-1* mutant than that of Col-0, respectively (Figure 2F). In response to Fe deficiency, *NAS2* was highly upregulated in Col-0 and all mutants except *fit1-2*. Interestingly, in response to Fe starvation, the upregulation was 2.1-fold and 3.4-fold higher in *sir1-1* and *cad2-1* mutant, respectively, compared with Col-0. S starvation did not have any effect on the expression of *NAS2* gene in all the tested lines. Moreover, in the case of dual nutrient starvation, the expression pattern of *NAS2* for all the lines was not significantly different compared to those observed under Fe starvation alone.

Fe homeostasis is tightly regulated to maintain the optimal Fe level in plants. *Arabidopsis* FERRIC REDUCTASE DEFECTIVE 3 (FRD3) is an important component of the iron homeostasis (Rogers and Guerinot, 2002; Green and Rogers, 2004). Although the transcript level of *FRD3* is only slightly regulated in response to Fe status (Rogers and Guerinot, 2002), we observed a significant accumulation of *FRD3* transcripts in the roots of all mutants compared to Col-0, under the conditions of Fe deficiency (Figure 2G). Remarkably, the lack of sulfite reduction in *sir1-1* and mutation in *GSH1* allele in *cad2-1* mutant constantly upregulated the expression of *FRD3* under nonstress conditions. In response to S starvation, a tendency of downregulation was observed in the transcript levels of *FRD3* in wild type and all mutant lines, compared to their respective normal growth conditions. Moreover, under dual Fe and S starvation, the expression of *FRD3* was significantly low in *fit1-2* and *sir1-1* mutants compared to their respective Fe starved conditions.

### Expression Analysis of Sulfate Transporter Genes

**Figure 3:**
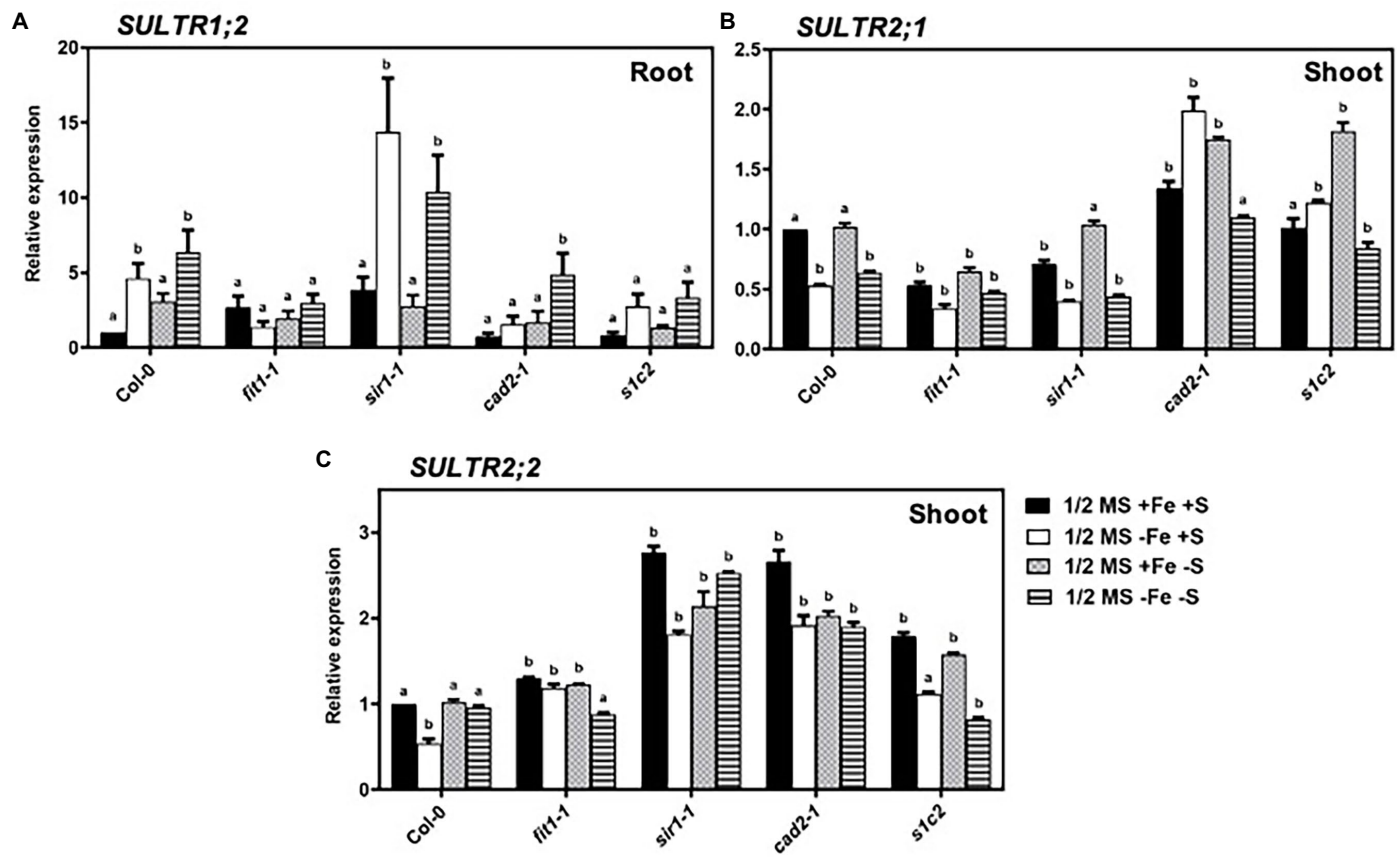
Expression profiles of sulfate transporter genes in *Arabidopsis*. Relative expression by Quantitative RT-PCR of the genes: **(A)** *SULTR1;2* in roots, **(B)** *SULTR2;1* in shoots, **(C)** *SULTR2;2* for *Arabidopsis* wild type (Col-0), *fit1-2*, *sir1-1*, *cad2-1*, and *s1c2* lines of 1-week old seedlings exposed to half-strength MS medium (1/2 MS + Fe + S), Fe-deficient (1/2 MS − Fe + S), S-deficient (1/2 MS + Fe − S), and Fe and S-deficient (1/2 MS − Fe − S) media for 4 days under long-day conditions. The *y*-axis shows RNA levels normalized to that of *GADPH*. **(A–C)** Letters indicate the statistically significant differences between the wild type (Col-0) on ½ MS + Fe + S with the other treatments in Col-0 and mutants determined with the two-way ANOVA test followed by Dunnett’s test (*p* < 0.05, *n* = 3). Bars represent means ± SD.

Expression patterns of the sulfate transporter genes from group 1 and group 2 were evaluated (Figure 3). The high-affinity sulfate transporter (*AtSULTR1;2*) is expressed predominately in roots and is responsible for the uptake of sulfate from soil solution into the root cells (Shibagaki et al., 2002; Yoshimoto et al., 2002). In response to Fe and S deficiency, the transcript of *sultr1;2* was 4.6-fold and 3-fold higher than that of Col-0 under the normal growth conditions, respectively (Figure 3A). In the *fit1-2* mutant, the expression of *AtSULTR1;2* tended to be higher than that of Col-0 under normal growth conditions. Interestingly, the upregulated expression of *AtSULTR1;2* under Fe deficiency was directly or indirectly dependent on *FIT* and *GSH1* because the upregulated expression of *AtSULTR1;2* disappeared in *fit1-2* and *cad2-1*. In the *cad2-1* mutant, *AtSULTR1;2* was significantly upregulated in response to dual nutrient starvation, but remained unchanged under the imposition of sole Fe or S starvation. The low-affinity sulfate transporters of group 2 are responsible for the translocation of sulfate within the plant. *AtSULTR1;2* and *AtSULTR2;2* are expressed throughout the plant in vascular tissues (Buchner et al., 2004). The expression of *AtSULTR1;2* was significantly downregulated under the sole Fe deficiency in the shoots of Col-0, and *sir1-1* (Figure 3B). However, contrary to the downregulated expression of *AtSULTR2;1* under Fe deficiency in Col-0, and *sir1-1*, the expression of *AtSULTR2;1* was rather significantly upregulated under Fe deficiency in the GSH-deficient *cad2-1*and *s1c2* mutants. It is noteworthy to mention that the lack of *FIT* in the *fit1-2* mutant, constantly downregulated the expression of *AtSULTR2;1*, irrespective of the nutrient status. Moreover, the expression of *AtSULTR2;1* remained unchanged in response to 4 days of S starvation in Col-0. Dual nutrient limitations triggered quite distinct and opposite responses compared to those observed under sole Fe or S deficiency in GSH-deficient mutants. The expression of *AtSULTR2;2* was significantly downregulated only in response to sole Fe deficiency but remained unchanged in response to 4 days of S or dual nutrient starvation in the shoots of Col-0. Strikingly, contrary to the expression of *AtSULTR2;1*, the lack of *FIT* in the *fit1-2* mutant constantly activated the expression of *AtSULTR2;2* under all experimental conditions except dual nutrient limitations (Figure 3C). Similarly, bottleneck in assimilatory sulfate reduction and GSH biosynthesis constantly activated the expression of *AtSULTR2;2* in the *sir1-1* and *cad2-1*mutant. The expression of *AtSULTR2;2* was maintained at a significantly higher level in *s1c2* mutant irrespective of the S status. However, its expression was downregulated under dual nutrient limitation, compared to Col-0.

### Expression Profiles of the Genes Related to Fe–S Cluster Biosynthesis in *Arabidopsis*

**Figure 4:**
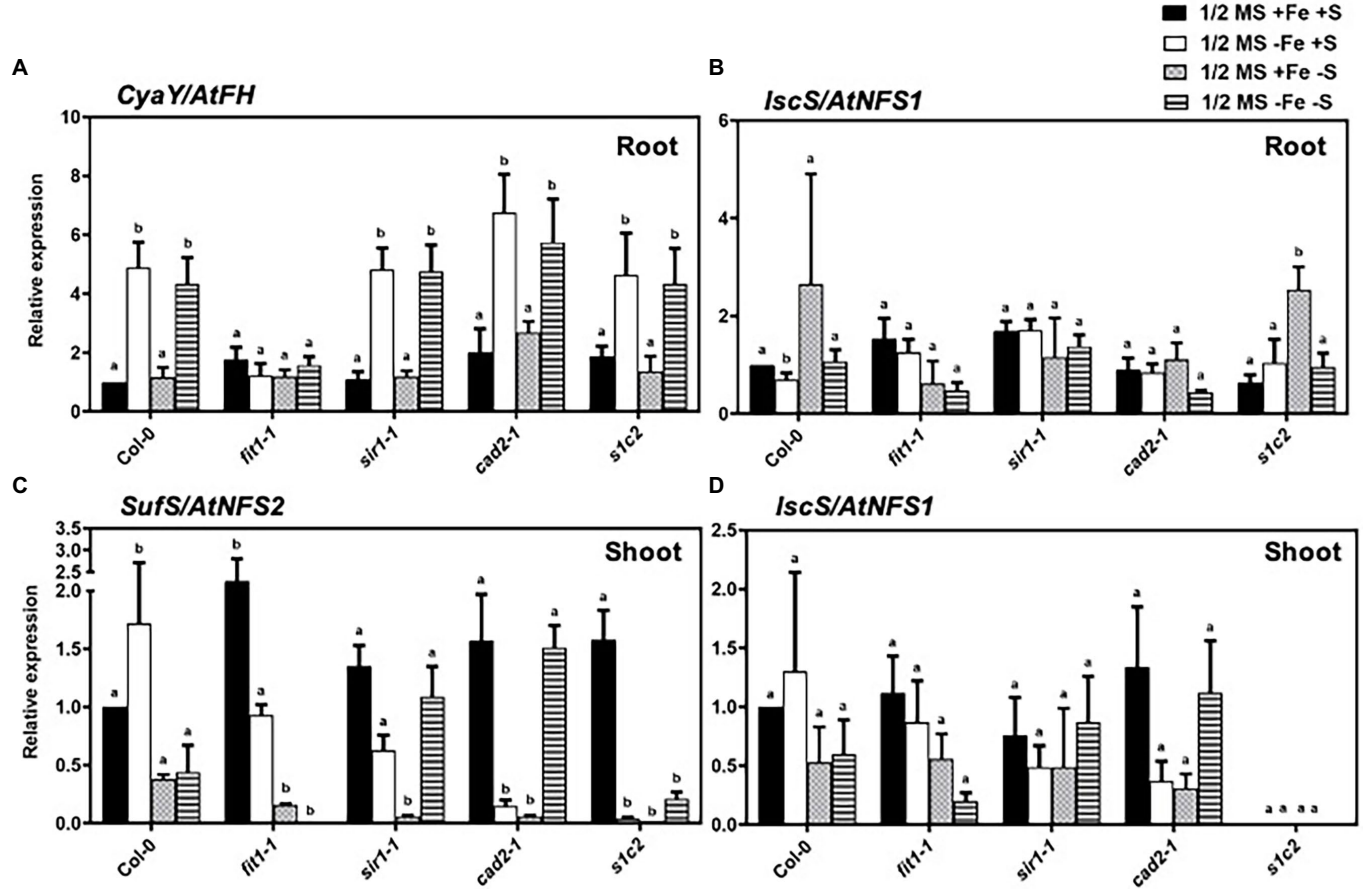
Expression profiles of the genes related to Fe–S cluster biosynthesis in *Arabidopsis*. Relative expression by Quantitative RT-PCR of the genes: **(A)** CyaY/*frataxin* in roots, **(B)** *IscS/NFS1* in roots, **(C)** *SufS/NFS2* in shoots and **(D)** *IscS/NFS1* in shoots, for *Arabidopsis* the wild type (Col-0), *fit1-2*, *sir1-1*, *cad2-1*, and *s1c2* lines of 1-week old seedlings exposed onto half-strength MS medium (1/2 MS + Fe + S), Fe-deficient (1/2 MS − Fe + S), S-deficient (1/2 MS + Fe − S) and Fe and S-deficient (1/2 MS − Fe − S) media for 4 days under long-day conditions. The *y*-axis shows RNA levels normalized to that of *GADPH*. **(A–D)** Letters indicate the statistically significant differences between the wild type (Col-0) on ½ MS + Fe + S with the other treatments in Col-0 and mutants determined with the two-way ANOVA test followed by Dunnett’s test (*p* < 0.05, *n* = 3). Bars represent means ± SD.

**Figure 5:**
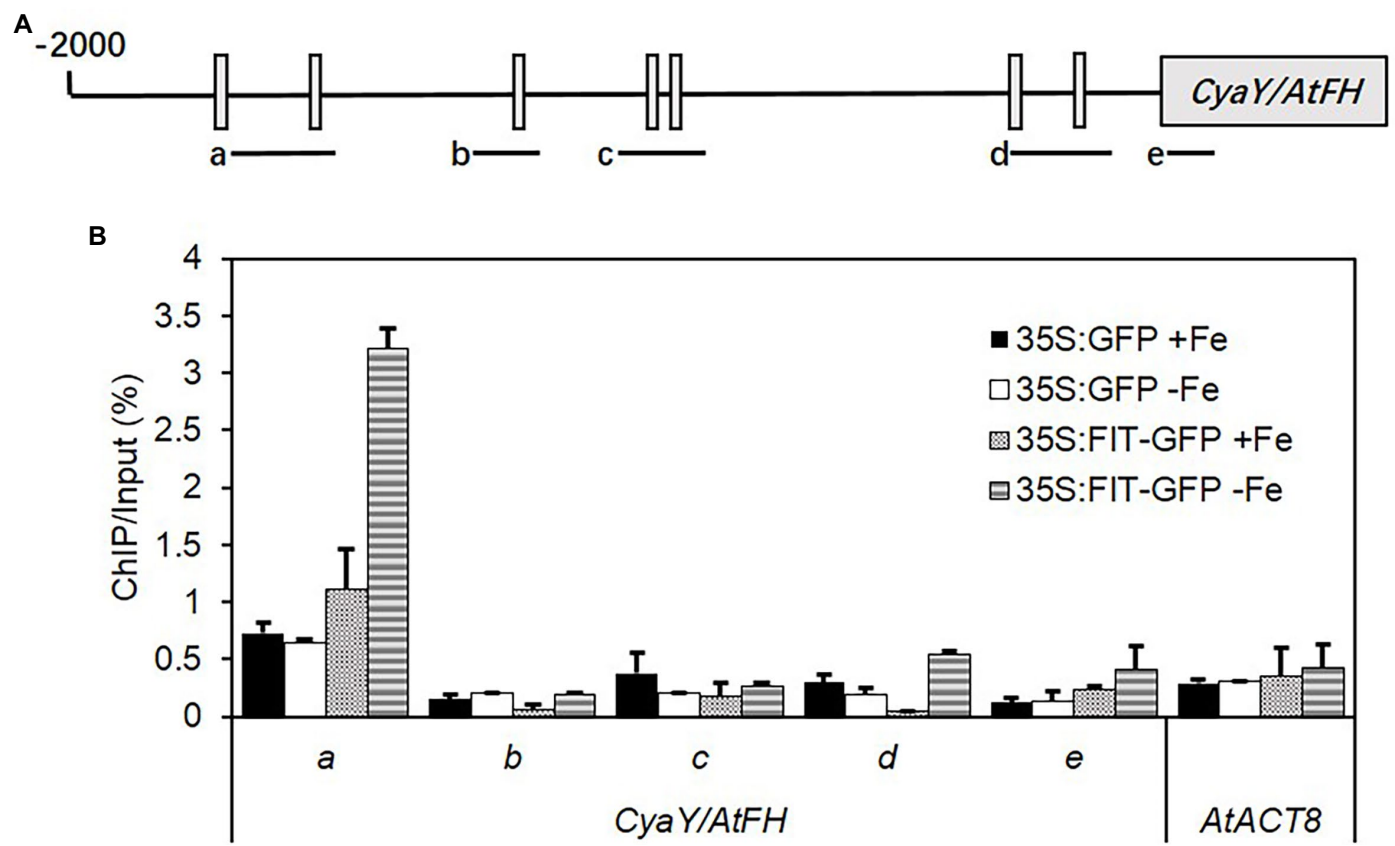
ChIP-qPCR analysis of the binding of FIT to the promoter of *AtFH*. Chromatin was extracted from 35S:FIT-GFP and 35S:GFP seedlings after administration of low iron stress for 7 days, and then precipitated using anti-GFP antibody. Precipitated DNA was amplified with primers corresponding to the different sequence regions of the *AtFH* promoters. **(A)** The ChIP signal obtained from multiple-quantitative ChIP-PCR was quantified as the percentage of total input DNA. **(B)** Three biological replicates were performed. Standard deviations were calculated from three technical repeats. Letters a–d indicate the amplified fragments from the promoter region of *AtFH*, respectively. ACTIN8 was used as a negative control.

CyaY/frataxin (annotated as *AtFH* in *Arabidopsis*) is an essential component for Fe–S cluster biosynthesis (Lill and Mühlenhoff, 2008; Stemmler et al., 2010), and plays a central role in regulating Fe homeostasis in mitochondria (Radisky et al., 1999; Bulteau et al., 2004). CyaY functions as Fe donor in the assembly of Fe–S (Klinge et al., 2007; Jain et al., 2014). The expression of *CyaY/AtFH* was induced by Fe and dual Fe and S deficiency in the roots of all lines (Figure 4A). However, the upregulated expression of *CyaY/AtFH* under Fe and dual Fe and S deficiency was directly or indirectly dependent on *FIT* because the upregulated expression of *CyaY/AtFH* disappeared in the *fit1-2* mutant. To further check the role of *FIT* in the regulation of *CyaY/AtFH* expression, we analyzed the expression profiles of *CyaY/AtFH* in the double overexpressor of *FIT* and its activating partner *bHLH38* (i.e., *OxFITXOXbHLH38*). *CyaY/AtFH* was constitutively overexpressed in the *OxFITXOXbHLH38* under nonstress conditions (Supplementary Figure S2). These experiments indicated that FIT might initiate *AtFH* expression through directly binding its promoter. We then performed ChIP-qPCR experiments to determine whether FIT directly interacts with the promoters of *AtFH*. Our ChIP-qPCR results support the *in vivo* binding of FIT to the promoters of *AtFH* (Figure 5). The deficiency of S alone did not cause any significant change in the expression *CyaY* in all the lines. In *Escherichia coli,* ISC and SUF are the two [Fe–S] biosynthesis systems. The group I cysteine desulfurase IscS (annotated as AtNFS1 in *Arabidopsis*) mobilizes S from cysteine in mitochondria for the biosynthesis of Fe–S clusters. The expression of *AtNFS1* did not change in response to sole Fe or S and dual deficiency of Fe and S in the shoots of all lines (Figure 4D). However, in the roots S deficiency did cause a significant upregulation of *AtNFS1* expression in *s1c2* mutant (Figure 4B). SufS (also annotated as AtNFS2 in *Arabidopsis*) is a group II cysteine desulfurase and is homologous to IscS/AtNFS1 in the plastids. The expression of *AtNFS2* was upregulated in the shoots of Col-0 under Fe deficiency (Figure 4C). The imposition of sole S starvation downregulated its expression in the shoots of all tested mutants compared to Col-0. It is noteworthy that in the GSH-deficient mutants (i.e., *cad2-1* and *s1c2*), sole Fe deficiency also significantly reduced its expression. Strikingly, in the *fit1-2* mutant, the expression of *AtNFS2* was upregulated under nonstress conditions.

## Discussion

The crosstalk between Fe and S at the physiological and molecular level has been documented in some early studies in different members of strategy I (Zuchi et al., 2009, 2015; Forieri et al., 2013, 2017; Muneer et al., 2014; Paolacci et al., 2014; Vigani et al., 2018) and strategy II plants (Astolfi et al., 2010; Ciaffi et al., 2013; Zamboni et al., 2017). The biogenesis of the Fe–S clusters is the best example of the interaction between Fe and S. The provision of reactive and potentially toxic components (Fe^2+^ and S^2−^) in defined stoichiometric ratios for the Fe–S clusters biogenesis requires a strict and multi-level control for their balanced acquisition and distribution within the plant to prevent toxicity. Growing evidence in recent years suggests dynamic crosstalk between Fe and S networks. To understand plant responses to the individual (Fe or S) and combined (Fe and S) nutrients limitations at the physiological and molecular level, we exposed *Arabidopsis thaliana* in wild type and different mutant backgrounds for 4 days to Fe, S, or combined Fe and S deficiencies. We observed that the GSH-deficient double mutant (*s1c2*) was highly sensitive to Fe deficiency compared to Col-0, *cad2-1,* and *sir1-1* mutants (Figure 1). The association of the endogenous GSH with tolerance to Fe deficiency has been suggested recently (Shanmugam et al., 2015). The GSH-deficient mutant *zir1,* which has about 15% of the wild-type GSH levels, was more sensitive to Fe deficiency compared to *pad2* (21% of the wild-type GSH levels), *cad2* (39% of the wild-type GSH levels), and Col-0 (Shanmugam et al., 2015). Since the GSH steady-state level of *s1c2* is indistinguishable from that of *cad2-1* (Speiser et al., 2018), the higher sensitivity of *s1c2*, compared to *cad2-1*, under Fe deficiency suggests that the Fe sensitive phenotype is not solely dependent on lower GSH contents as assumed earlier (Shanmugam et al., 2015). This is further supported by the observation, that despite the significantly higher GSH levels (Speiser et al., 2018), the *sir1-1* mutant was even slightly more sensitive than *cad2-1* (Figure 1; Supplementary Figure S1). The sensitivity of the GSH-deficient mutant under Fe limitation is therefore intriguing and is not solely dependent on the endogenous level of total GSH or the amount of soluble protein-bound S, as the amount of soluble protein-bound S in the *s1c2* was significantly higher than *sir1-1* but lower than *cad2-1* under normal growth conditions (Speiser et al., 2018). Although other possibilities could not be ruled out, the sensitivity of *s1c2* could partially be linked to the more oxidized state of GSH in the plastids of *s1c2* compared to *cad2-1*, *sir1-1,* and Col-0 as demonstrated in the same study. Alternatively, all these factors (i.e., redox state of GSH in plastids, amount of soluble protein-bound S, and endogenous level of total GSH) may have an overall additive effect in modulating plant responses under Fe limited conditions.

We also observed that irrespective of the mutant background, the growth of seedlings was negatively affected under S deprivation, but they all looked greener compared to the seedlings grown under Fe deficiency. The latter observation is not in agreement with the previous studies reporting that S deficiency induces chlorosis in strategy II plant species (Ciaffi et al., 2013; Zamboni et al., 2017). The Fe deficiency symptoms (chlorosis) induced under S deficiency can be attributed to the inability of the strategy II plants to synthesize the S-derived phytosiderophores that are required for Fe uptake (Mori and Nishizawa, 1987). In addition to the basic differences between strategy I (reduction-based Fe uptake) and strategy II (chelation-based Fe uptake) plants, additional levels of regulation may exist to fine-tune plant’s response to Fe or S deprivation (Mendoza-Cózatl et al., 2019). The less pronounced chlorosis in *Arabidopsis* plants exposed to dual deficiency of Fe and S compared to plants exposed to sole Fe starvation has been recently reported (Robe et al., 2020). These authors have suggested that experimental conditions and species- or developmental stage-specific (i.e., seedlings vs. mature plants, young vs. mature leaves) mechanisms may be modulating plant response to the individual (S or Fe) and combined (Fe and S) nutrient starvation.

While the upregulation of *IRT1* in response to Fe deficiency (Forieri et al., 2013; Muneer et al., 2014; Zuchi et al., 2015) and S deficiency (Muneer et al., 2014; Zuchi et al., 2015) has been previously reported, here we demonstrated that the *cad2-1* allelic mutation of *GSH1* (γ-glutamylcysteine synthetase) gene overrode the typical signal of *IRT1* upregulation in the *cad2-1* and *s1c2* mutants under S deficiency. Moreover, the bottleneck in sulfate assimilation (Khan et al., 2010) due to *SIR* knockdown mutation constitutively upregulated *IRT1* expression in the *sir1-1* mutant under all experimental conditions.

The induction of *FRO2* in response to Fe deficiency is well documented (Muneer et al., 2014; Zuchi et al., 2015; Forieri et al., 2017). However, in addition to sole Fe starvation, S deficiency also upregulated the expression of *FRO2* in Col-0 seedlings compared to control conditions. These findings are consistent with the previous reports (Muneer et al., 2014; Zuchi et al., 2015). However, the upregulated *FRO2* expression under S deficiency disappeared in the *sir1-1*, *cad2-1,* and *s1c2,* suggesting that the expression of *FRO2* may be directly or indirectly dependent on *GSH1* and/or *SIR.* Moreover, given the fact that the *sir1-1*, *cad2-1*, and *s1c2* seedlings are already suffering from S deficiency even under normal growth conditions (Speiser et al., 2018), the reduced expression of *FRO2* in the *sir1-1*, *cad2-1*, and *s1c2* seedlings compared to Col-0 suggest that under severe/prolonged S starvation, the expression of *FRO2* is downregulated as a consequence of secondary adaptations. It is noteworthy that contrary to the expression pattern of *IRT1*, the expression of *FRO2* was not constitutively upregulated in the *sir1-1* mutant. The differential expression of *IRT1* and expression of *FRO2* under S deficiency in the *sir1-1* mutant suggests that some other unknown signals in the *sir1-1* mutant might be triggering the expression of *IRT1*.

The induction of *FIT* in response to Fe deficiency is well known (Colangelo and Guerinot, 2004). FIT-dependent protein complexes through hetero-dimerization with clade Ib bHLHs (e.g., bHLH38) are required for the activation of the expression of genes involved in the maintenance of Fe homeostasis such as *IRT1* and *FRO2*. We observed similar expression patterns (induction in response to sole Fe or combined S and Fe starvation) for *FIT* and *bHLH38* in our study. The upregulation of *FIT* and *bHLH38* upon Fe starvation is consistent with the previous reports (Colangelo and Guerinot, 2004; Ivanov et al., 2012). Moreover, the upregulation of *bHLH38* in the *fit1-2* mutant corroborates the previous findings that the expression of the clade Ib bHLH genes (i.e., *bHLH38*, *bHLH39*, *bHLH100*, and *bHLH101*) is not dependent on *FIT* and that the expression of *FIT* and *bHLH38* is controlled by different pathways, presumably by different signals upon Fe starvation (Wang et al., 2007). Unlike the expression pattern of *IRT1* and *FRO2*, the expression of *FIT* and *bHLH38* did not change under S deficiency in Col-0 and the *sir1-1*, *cad2-1*, and *s1c2*. It is also worth mentioning that although these expression profiles are consistent with many of the previous reports, however, under different experimental sets up opposite expression patterns, i.e., repression of *IRT1* (Forieri et al., 2013), *FRO2*, and *FIT* (Forieri et al., 2017) under S starvation have also been reported. Different growth conditions, durations of the nutrient starvations (4 days starvation in this study vs. 5 weeks starvation) and developmental/stage-specific (i.e., seedlings vs. mature plants) mechanisms may account for these differences. Moreover, contrary to (Forieri et al., 2013, 2017), the presence of sucrose; the end product of photosynthesis, in growth medium in our experiments can expected to alter plant’s responses to Fe or S deprivation.

Mounting evidence suggests critical roles for GSH in cell signaling (Zhang and Forman, 2012) and its cross-communication with other established signaling molecules (Ghanta and Chattopadhyay, 2011). A variety of signaling pathways are involved in the regulation of *GSH1* (reviewed in Zhang and Forman, 2012). Therefore, it remains to be elucidated whether the differential regulation of *IRT1* and *FRO2* under S deficiency is due to mutations in the *GSH1* gene in the *cad2-1* mutant or GSH itself acts as a major player in regulating their expression. The ROS dependent regulation of Fe homeostasis related genes has been recently reported in several studies (Astolfi et al., 2021; von der Mark et al., 2021; McInturf et al., 2022). Although other possibilities could not be ruled out, the non-canonical responses of some of the known marker genes of Fe homeostasis in GSH deficient mutants suggest that presumably, the elevated ROS levels in these mutants might be responsible for the differential regulation of these genes.

*NAS1*, *NAS2*, and *FRD3* are key genes involved in the transport of Fe through the phloem and xylem conducting tissues, respectively. In line with the previous reports, the expression of *NAS1* and *NAS2* was upregulated under Fe-starved conditions to enhance the translocation of Fe (Kim et al., 2005; Klatte et al., 2009). S deficiency did not cause any significant changes in the expression of these two genes, which is contrary to the previous report showing S deficiency completely blocked the expression of the NA synthase gene (Zuchi et al., 2009) and NA accumulation (Zuchi et al., 2015) in tomato independently from the availability of Fe. The observed discrepancy might be linked to the different threshold levels of S deficiency perceived by plants or different mechanisms operating in the *Arabidopsis* model system and tomato as described previously. The expression of *NAS1* and *NAS2* in our study was rather upregulated under S starvation in the GSH-deficient *cad2-1* but not in *s1c2* mutant. Irrespective of the availability of S, the deficiency of Fe caused slight to moderate induction in the expression of *FRD3* in all lines which corroborates previous findings (Rogers and Guerinot, 2002). Interestingly, in wild type and all mutant lines the transcripts of *FRD3* tended to be low (although non-significantly) under S starvation, which may be an adaptation to the lowered need of the partner nutrient Fe for Fe–S clusters biosynthesis. Under nonstress conditions, the lack of sulfite reduction in *sir1-1* and *cad2-1* allelic mutation in *GSH1* gene constantly upregulated the expression of *FRD3*.

The uptake and subsequent assimilation of sulfate are dependent both on plant S demand for growth and external S supply (Hawkesford and De Kok, 2006). The expression of the high-affinity group 1 sulfate transporter (*AtSULTR1;2*), that is responsible for the primary uptake of sulfate by the root (Buchner et al., 2010), was upregulated in response to S deficiency in agreement with previous reports (Lewandowska and Sirko, 2008; Takahashi et al., 2011; Zuchi et al., 2015; Forieri et al., 2017). Remarkably, there was a significant increase in the *AtSULTR1;2* transcript abundance after imposition of sole Fe deficiency. This is consistent to the expression pattern of *AtSULTR1;2* in tomato (Zuchi et al., 2015), and increased total S concentration in tomato (Paolacci et al., 2014) and wheat (Ciaffi et al., 2013) plants exposed to short term Fe starvation like our experimental conditions, but opposite to what has been reported in *Arabidopsis* in response to long term (4–11 days vs. 5 weeks) Fe starvation (Forieri et al., 2017). The upregulated expression of *AtSULTR1;2* disappeared in the mutant *fit1-2*, which suggest that under Fe deficiency, the expression of *AtSULTR1;2* may be directly or indirectly dependent on *FIT*. Alternatively, these observations also suggest that perception of Fe starvation signals beyond certain threshold level leads to secondary adaptations to adjust plants demands to changing requirement of the partner nutrient S. This is supported by the fact that in the *fit1-2* mutant, which is already suffering from Fe starvation even under normal Fe supply, the expression pattern of *AtSULTR1;2* exhibited an opposite but comparable trend to what has reported in *Arabidopsis* in response to long-term Fe starvation (Forieri et al., 2017).

In contrast to the expression pattern of the high-affinity group 1 sulfate transporter (*AtSULTR1;2*) in the root, the low-affinity sulfate transporters (*AtSULTR2;1* and *AtSULTR2;2*) that are responsible for translocation of sulfate within the plant (Takahashi et al., 2000) showed a different expression pattern in the shoots (Figures 3B,C). First, short-term exposure (4 days) to S starvation did not change the expression of these two low-affinity sulfate transporters in Col-0, which is not consistent with the previous reports showing their upregulation in response to medium term (10 days) S starvation (Zuchi et al., 2015). Secondly, in contrast to the significant upregulation of the high affinity sulfate transporter, sole Fe starvation significantly lowered the expression of *AtSULTR2;1* and *AtSULTR2;2* in Col-0. These observations suggest that Fe deficiency signals are perceived and integrated earlier in plant’s responses compared to S. However, contrary to the downregulated expression of *AtSULTR2;1* under Fe deficiency in Col-0, the expression of *AtSULTR2;1* was significantly upregulated in the GSH-deficient *cad2-1* and *s1c2* mutants, which suggest that GSH might act as a signaling molecule to modulate the expression of *AtSULTR2;1*. It is noteworthy to mention that the lack of *FIT* in the *fit1-2* mutant, constantly downregulated the expression of *AtSULTR2;1*, irrespective of the nutrient status. It is noteworthy that in the *sir1-1* and *fit1-2* mutants, which are already sufferings from S and Fe starvation, respectively, even under natural conditions, the expression of *AtSULTR2;1* was kept at a significantly lower level compared to Col-0. However, the expression of *AtSULTR2;2* in the *sir1-1* and *fit1-2* mutants under unstress conditions was significantly high compared to Col-0 (Figure 3C). Such contrasting differences in the expression of *AtSULTR2;1* and *AtSULTR2;2* suggest different functions for these two transporters in plants exposed to long term S or Fe deficiency. Similarly, like *sir1-1* mutant, bottleneck in GSH biosynthesis also constantly upregulated the expression of *AtSULTR2;2* in the *cad2-1* mutant under all experimental conditions. *AtSULTR2;1* and *AtSULTR2;2* tend to express in vascular tissues throughout the plant (Buchner et al., 2004). *AtSULTR2;1* is believed to be responsible for uptake of S from the apoplasm within the vascular bundle and involved in root to shoot transport (Takahashi et al., 2000). Our data suggest that the reduced uptake of sulfate from the apoplast is one of the adaptation mechanisms to adjust S requirements under Fe deficiency. The adjustment of S uptake and assimilation as a Fe deficiency adaptation has been previously demonstrated (Astolfi et al., 2006; Ciaffi et al., 2013; Paolacci et al., 2014). Our work indicates that dual limitation of Fe and S can trigger quite distinct responses compared to sole Fe or S limitations, especially in the background of *cad2-1* mutant. For example, *AtSULTR1;2* was significantly upregulated in response to dual nutrient starvation, but remained unchanged under the imposition of sole Fe or S starvation in the *cad2-1* mutant. Similarly, the expression of *AtSULTR2;1* (in *cad2-1* and *s1c2*) and *AtSULTR2;2* (in *s1c2*) under dual nutrient limitations showed an opposite trend compared to sole Fe or S starved conditions. These observations underlined the significance of understanding the underlying mechanisms that govern plant responses under dual nutrient limitation to figure out strategies for sustainable plant growth in nutrient-limited environments.

The reciprocal influence between Fe and S in terms of uptake, transport, and assimilation has been demonstrated in many studies (Astolfi et al., 2006; Zuchi et al., 2009, 2015; Ciaffi et al., 2013; Forieri et al., 2013, 2017; Paolacci et al., 2014). However, the crosstalk between Fe and S in terms of regulation of genes involved in the biosynthesis of Fe–S clusters has been rarely investigated, despite the fact that the major portion of the metabolically active Fe is bound to S in the form of Fe–S clusters. Fe and S interact for the building of Fe–S assembly, and three types of machinery for Fe–S cluster assembly have been identified and are distributed in the cytosol (CIA for cytosolic Fe–S protein assembly), the mitochondria (ISC for Fe–S cluster) and the chloroplasts (SUF for S mobilization) in plants (Lill and Mühlenhoff, 2008). Since mitochondria are one of the cellular compartments where the Fe–S cluster assembly takes place. Therefore, mitochondria might be expected to play a central role in the regulation of Fe and S for the Fe–S cluster assembly. In mitochondria, frataxin (FH) plays a pivotal role in regulating Fe homeostasis (Radisky et al., 1999; Bulteau et al., 2004) and Fe–S clusters biosynthesis (Lill and Mühlenhoff, 2008; Stemmler et al., 2010). The significant upregulation of *FH* in the roots of Col-0 and all mutants only in response to sole Fe or dual Fe and S deficiency suggests that the S status of the plant has apparently no role in modulating the expression of *FH*. Quite interestingly, the up-regulated expression of *FH* under Fe deficiency is directly or indirectly dependent on *FIT* because the upregulated expression of *FH* disappeared in the *fit1-2* mutant. The constitutive upregulation of *FH* expression in the double overexpresser of *FIT* and its activating partner *bHLH38* (i.e., *OxFITXOxbHLH38*) under nonstress conditions and our ChIP-qPCR experiments supports *in vivo* binding of FIT to the promoters of *AtFH.* This is an exciting new prospect for future research and additional evidence is required to validate this hypothesis. In mitochondria and plastids, the required S atoms for the assembly of Fe–S are extracted from cysteine by pyridoxal phosphate-dependent cysteine desulfurases, IscS/NFS1, and SufS/NFS2, respectively (Couturier et al., 2013). *NFS2* is specifically adapted to oxidative stress and Fe starvation (Stemmler et al., 2010). Interestingly, the expression of *NFS2* was significantly upregulated in response to Fe deficiency in the shoots of Col-0 seedlings but dramatically reduced in response to S starvation in all the mutants and to a lesser extent in Col-0. Moreover, in the *fit1-2* and the rest of the mutants, which are already suffering from Fe and S starvation, respectively, the deficiency of Fe either down-regulated (*cad2-1* and *s1c2*) or did not change (*fit1-2* and *sir1-1*) the expression of *NFS2*. Overall, these findings suggest that in terms of Fe–S clusters biogenesis, major adaptations in response to Fe deficiency occur in mitochondria (*via* regulation of *FH*), whereas adaptations to S deficiency are mainly taking place in plastids (*via* regulation of *NFS2*), to rebalance overall requirements of the plants for the two partner nutrients.

**Figure 6:**
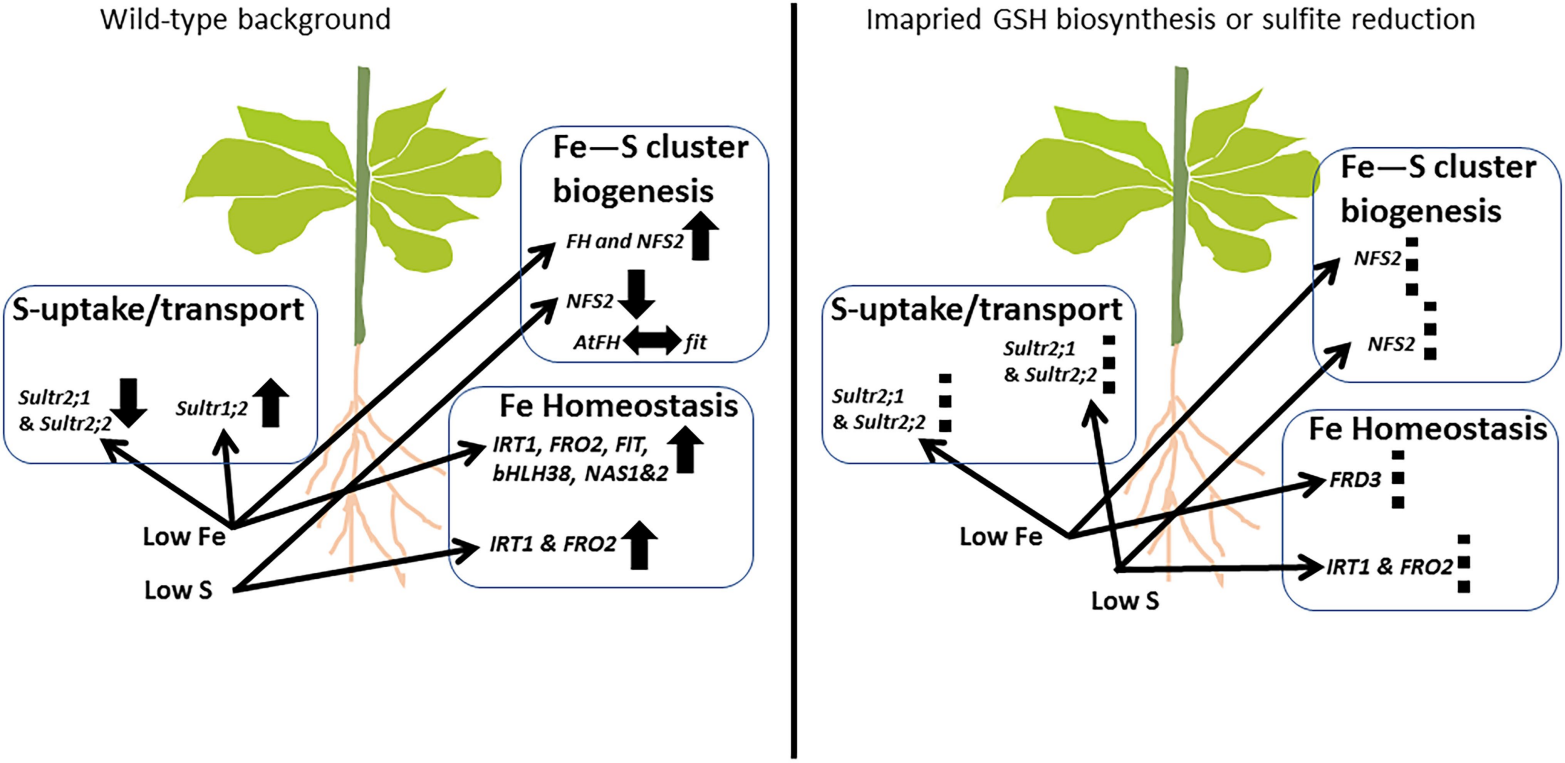
Schematic representation of the interplay between iron and sulfur networks in *Arabidopsis*. The availability of Fe and S affect the expression of S and Fe homeostasis related gene in plants. The response pattern might depend on the severity/degree of nutrient deficiency. Bottlenecks in sulfite reduction and/or GSH biosynthesis (**right panel**) can futher modulate the cannonical responses *via* still unknown signals like ROS (Astolfi et al., 2021; von der Mark et al., 2021; McInturf et al., 2022) and others. UP arrows: Upregulation; down arrows: downregulation; left–right arrow: probable interaction; and dashed-lines: differential regulation compared to wild-type background.

Our findings related to Fe sensitivity of the GSH-deficient mutants add significant new insights to the existing knowledge, linking the endogenous level of total GSH to Fe sensitivity under Fe limitation. The relative sensitivity of *sir1-1*, *cad2-1*, *s1c2* under Fe starvation suggests that Fe sensitivity is not solely dependent on the endogenous level of total GSH as hypothesized earlier (Shanmugam et al., 2015). We demonstrated that the known reciprocal signals between iron and sulfur networks are differentially regulated by bottlenecks in sulfite reduction and/or GSH biosynthesis (Figure 6). We also demonstrated that bottlenecks in sulfite reduction and/or GSH biosynthesis differentially regulate the expression of some of the known marker genes of Fe homeostasis, S uptake/transport, and Fe–S clusters biogenesis. We also elucidated the crosstalk between Fe and S in terms of expression of some of the key players involved in the assembly of Fe–S clusters biogenesis machinery in the plastids and mitochondria. We observed major adaptations in the mitochondria in response to Fe deficiency and the plastids in response to S deficiency. Our data suggest that *FH* is a potential target of FIT. While the dual limitation of Fe and S mostly generated overruling and/or synergistic signals, quite distinct responses, as opposed to sole Fe or S limitations, were also observed. These observations underscored the significance of elucidating plant responses under dual nutrient limitations for sustainable plant growth in nutrient-limited environments.

## Data Availability Statement

The original contributions presented in the study are included in the article/Supplementary Material, further inquiries can be directed to the corresponding authors.

## Author Contributions

H-QL, TC, and HW planned and designed the research. MSK, QL, MC, and HR performed experiments and analyzed data. MSK and H-QL wrote the manuscript. All authors contributed to the article and approved the submitted version.

## Conflict of Interest

The authors declare that the research was conducted in the absence of any commercial or financial relationships that could be construed as a potential conflict of interest.

## Publisher’s Note

All claims expressed in this article are solely those of the authors and do not necessarily represent those of their affiliated organizations, or those of the publisher, the editors and the reviewers. Any product that may be evaluated in this article, or claim that may be made by its manufacturer, is not guaranteed or endorsed by the publisher.

## Supplementary Material

Supplementary Figure S1 Phenotypes of 14-days old seedlings under Fe-deficient conditions. Top view of the seedlings directly germinated on Fe-deficient half-strength MS medium for 14 days.

Supplementary Figure S2 Expression analysis of *AtFH* in the double overexpressor *OxFIT X OxbHLH38*. Relative expression by Quantitative RT-PCR of the genes*CyaY/frataxin* in roots,for *Arabidopsis* the wildtype (Col-0) and the double overexpressor line *OxFITXOxbHLH38* of one-week old seedlings exposed to half-strength MS medium (1/2 MS +Fe +S), Fe deficient (1/2 MS -Fe +S), S deficient (1/2 MS +Fe -S) and Fe & S deficient (1/2 MS –Fe -S) media for four days under long-day conditions. The y-axis shows RNA levels normalized to that of GADPH. Letters indicate the statistically significant differences among all treatments in Col-0 and doubleoverxpresser line determined with the two-way ANOVA test followed by Tukey’s test (P < 0.05, n = 3). Bars represent means ± SD.

## References

[ref1] Astolfi S. Celletti S. Vigani G. Mimmo T. Cesco S. (2021). Interaction between sulfur and iron in plants. Front. Plant Sci. 12:670308. doi: 10.3389/fpls.2021.670308, PMID: 34354720 PMC8329491

[ref2] Astolfi S. Cesco S. Zuchi S. Neumann G. Roemheld V. (2006). Sulfur starvation reduces phytosiderophores release by iron-deficient barley plants. Soil Sci. Plant Nutr. 52, 43–48. doi: 10.1111/j.1747-0765.2006.00010.x

[ref3] Astolfi S. Zuchi S. Hubberten H.-M. Pinton R. Hoefgen R. (2010). Supply of Sulphur to S-deficient young barley seedlings restores their capability to cope with iron shortage. J. Exp. Bot. 61, 799–806. doi: 10.1093/jxb/erp346, PMID: 20018904 PMC2814111

[ref4] Bauer P. Ling H.-Q. Guerinot M. L. (2007). *FIT*, the *FER*-like iron deficiency induced transcription factor in *Arabidopsis*. Plant Physiol. Biochem. 45, 260–261. doi: 10.1016/j.plaphy.2007.03.006 17466530

[ref01] Bowler C. Benvenuto G. Laflamme P. Molino D. Probst A. V. Tariq M.. (2004). Chromatin techniques for plant cells. Plant J. 39, 776–789. doi: 10.1111/j.1365-313X.2004.02169.x 15315638

[ref5] Buchner P. Parmar S. Kriegel A. Carpentier M. Hawkesford M. J. (2010). The sulfate transporter family in wheat: tissue-specific gene expression in relation to nutrition. Mol. Plant 3, 374–389. doi: 10.1093/mp/ssp119, PMID: 20118181

[ref6] Buchner P. Stuiver C. E. E. Westerman S. Wirtz M. Hell R. Hawkesford M. J.. (2004). Regulation of sulfate uptake and expression of sulfate transporter genes in *Brassica oleracea* as affected by atmospheric H2S and pedospheric sulfate nutrition. Plant Physiol. 136, 3396–3408. doi: 10.1104/pp.104.046441, PMID: 15377780 PMC523398

[ref7] Bulteau A.-L. O'Neill H. A. Kennedy M. C. Ikeda-Saito M. Isaya G. Szweda L. I. (2004). Frataxin acts as an iron chaperone protein to modulate mitochondrial aconitase activity. Science 305, 242–245. doi: 10.1126/science.1098991, PMID: 15247478

[ref8] Ciaffi M. Paolacci A. R. Celletti S. Catarcione G. Kopriva S. Astolfi S. (2013). Transcriptional and physiological changes in the S assimilation pathway due to single or combined S and Fe deprivation in durum wheat (*Triticum durum* L.) seedlings. J. Exp. Bot. 64, 1663–1675. doi: 10.1093/jxb/ert027, PMID: 23390290 PMC3617832

[ref9] Cobbett C. S. May M. J. Howden R. Rolls B. (1998). The glutathione-deficient, cadmium-sensitive mutant, cad2–1, of *Arabidopsis thaliana* is deficient in γ-glutamylcysteine synthetase. Plant J. 16, 73–78. doi: 10.1046/j.1365-313x.1998.00262.x, PMID: 9807829

[ref10] Colangelo E. P. Guerinot M. L. (2004). The essential basic helix-loop-helix protein FIT1 is required for the iron deficiency response. Plant Cell 16, 3400–3412. doi: 10.1105/tpc.104.024315, PMID: 15539473 PMC535881

[ref11] Connolly E. L. Campbell N. H. Grotz N. Prichard C. L. Guerinot M. L. (2003). Overexpression of the FRO2 ferric chelate reductase confers tolerance to growth on low iron and uncovers posttranscriptional control. Plant Physiol. 133, 1102–1110. doi: 10.1104/pp.103.025122, PMID: 14526117 PMC281606

[ref12] Connolly E. L. Guerinot M. L. (2002). Iron stress in plants. Genome Biol. 3, 1021–1024. doi: 10.1186/gb-2002-3-8-reviews1024, PMID: 12186653 PMC139400

[ref13] Couturier J. Touraine B. Briat J.-F. Gaymard F. Rouhier N. (2013). The iron-sulfur cluster assembly machineries in plants: current knowledge and open questions. Front. Plant Sci. 4:259. doi: 10.3389/fpls.2013.00259, PMID: 23898337 PMC3721309

[ref14] Fan H. Zhang Z. Wang N. Cui Y. Sun H. Liu Y.. (2014). SKB1/PRMT 5-mediated histone H 4 R 3 dimethylation of I b subgroup bHLH genes negatively regulates iron homeostasis in *Arabidopsis thaliana*. Plant J. 77, 209–221. doi: 10.1111/tpj.12380, PMID: 24298997

[ref15] Forieri I. Sticht C. Reichelt M. Gretz N. Hawkesford M. J. Malagoli M.. (2017). System analysis of metabolism and the transcriptome in *Arabidopsis thaliana* roots reveals differential co-regulation upon iron, sulfur and potassium deficiency. Plant Cell Environ. 40, 95–107. doi: 10.1111/pce.12842 27726154

[ref16] Forieri I. Wirtz M. Hell R. (2013). Toward new perspectives on the interaction of iron and sulfur metabolism in plants. Front. Plant Sci. 4:357. doi: 10.3389/fpls.2013.00357, PMID: 24106494 PMC3788360

[ref17] Ghanta S. Chattopadhyay S. (2011). Glutathione as a signaling molecule-another challenge to pathogens: another challenge to pathogens. Plant Signal. Behav. 6, 783–788. doi: 10.4161/psb.6.6.15147, PMID: 21969955 PMC3218473

[ref18] Green L. S. Rogers E. E. (2004). FRD3 controls iron localization in *Arabidopsis*. Plant Physiol. 136, 2523–2531. doi: 10.1104/pp.104.045633, PMID: 15310833 PMC523319

[ref19] Hawkesford M. J. De Kok L. J. (2006). Managing sulphur metabolism in plants. Plant Cell Environ. 29, 382–395. doi: 10.1111/j.1365-3040.2005.01470.x, PMID: 17080593

[ref20] Hirai M. Y. Fujiwara T. Awazuhara M. Kimura T. Noji M. Saito K. (2003). Global expression profiling of sulfur-starved *Arabidopsis* by DNA macroarray reveals the role of O-acetyl-l-serine as a general regulator of gene expression in response to sulfur nutrition. Plant J. 33, 651–663. doi: 10.1046/j.1365-313X.2003.01658.x, PMID: 12609039

[ref21] Ivanov R. Brumbarova T. Bauer P. (2012). Fitting into the harsh reality: regulation of iron-deficiency responses in dicotyledonous plants. Mol. Plant 5, 27–42. doi: 10.1093/mp/ssr065, PMID: 21873619

[ref22] Jain A. Wilson G. T. Connolly E. L. (2014). The diverse roles of FRO family metalloreductases in iron and copper homeostasis. Front. Plant Sci. 5:100. doi: 10.3389/fpls.2014.00100, PMID: 24711810 PMC3968747

[ref23] Jeffrey S. W. Humphrey G. F. (1975). New spectrophotometric equations for determining chlorophylls a, b, c1 and c2 in higher-plants, algae and natural phytoplankton. Biochem. Physiol. Pflanz. 167, 191–194. doi: 10.1016/S0015-3796(17)30778-3

[ref24] Khan M. S. Haas F. H. Samami A. A. Gholami A. M. Bauer A. Fellenberg K.. (2010). Sulfite reductase defines a newly discovered bottleneck for assimilatory sulfate reduction and is essential for growth and development in *Arabidopsis thaliana*. Plant Cell 22, 1216–1231. doi: 10.1105/tpc.110.074088, PMID: 20424176 PMC2879758

[ref25] Kim S. Takahashi M. Higuchi K. Tsunoda K. Nakanishi H. Yoshimura E.. (2005). Increased nicotianamine biosynthesis confers enhanced tolerance of high levels of metals, in particular nickel, to plants. Plant Cell Physiol. 46, 1809–1818. doi: 10.1093/pcp/pci196, PMID: 16143596

[ref26] Klatte M. Schuler M. Wirtz M. Fink-Straube C. Hell R. Bauer P. (2009). The analysis of *Arabidopsis* nicotianamine synthase mutants reveals functions for nicotianamine in seed iron loading and iron deficiency responses. Plant Physiol. 150, 257–271. doi: 10.1104/pp.109.136374, PMID: 19304929 PMC2675739

[ref27] Klinge S. Hirst J. Maman J. D. Krude T. Pellegrini L. (2007). An iron-sulfur domain of the eukaryotic primase is essential for RNA primer synthesis. Nat. Struct. Mol. Biol. 14, 875–877. doi: 10.1038/nsmb1288, PMID: 17704817 PMC2268749

[ref28] Lewandowska M. Sirko A. (2008). Recent advances in understanding plant response to sulfur-deficiency stress. Acta Biochim. Pol. 55, 457–471. doi: 10.18388/abp.2008_3051, PMID: 18787711

[ref29] Lill R. Mühlenhoff U. (2008). Maturation of iron-sulfur proteins in eukaryotes: mechanisms, connected processes, and diseases. Annu. Rev. Biochem. 77, 669–700. doi: 10.1146/annurev.biochem.76.052705.162653, PMID: 18366324

[ref30] McGrath S. P. Zhao F. Blake-Kalff M. (2003). History and outlook for sulphur fertilizers in Europe. Ferti. Fertilization 2, 5–27.

[ref31] McInturf S. A. Khan M. A. Gokul A. Castro-Guerrero N. A. Höhner R. Li J.. (2022). Cadmium interference with iron sensing reveals transcriptional programs sensitive and insensitive to reactive oxygen species. J. Exp. Bot. 73, 324–338. doi: 10.1093/jxb/erab393, PMID: 34499172

[ref32] Mendoza-Cózatl D. G. Gokul A. Carelse M. F. Jobe T. O. Long T. A. Keyster M. (2019). Keep talking: crosstalk between iron and sulfur networks fine-tunes growth and development to promote survival under iron limitation. J. Exp. Bot. 70, 4197–4210. doi: 10.1093/jxb/erz290, PMID: 31231775

[ref33] Mori S. Nishizawa N. (1987). Methionine as a dominant precursor of phytosiderophores in Graminaceae plants. Plant Cell Physiol. 28, 1081–1092.

[ref34] Muneer S. Lee B.-R. Kim K.-Y. Park S.-H. Zhang Q. Kim T.-H. (2014). Involvement of Sulphur nutrition in modulating iron deficiency responses in photosynthetic organelles of oilseed rape (*Brassica napus* L.). Photosynth. Res. 119, 319–329. doi: 10.1007/s11120-013-9953-8, PMID: 24264737

[ref35] Nikiforova V. J. Kopka J. Tolstikov V. Fiehn O. Hopkins L. Hawkesford M. J.. (2005). Systems rebalancing of metabolism in response to sulfur deprivation, as revealed by metabolome analysis of *Arabidopsis* plants. Plant Physiol. 138, 304–318. doi: 10.1104/pp.104.053793, PMID: 15834012 PMC1104185

[ref36] Paolacci A. R. Celletti S. Catarcione G. Hawkesford M. J. Astolfi S. Ciaffi M. (2014). Iron deprivation results in a rapid but not sustained increase of the expression of genes involved in iron metabolism and sulfate uptake in tomato (*Solanum lycopersicum* L.) seedlings. J. Integr. Plant Biol. 56, 88–100. doi: 10.1111/jipb.12110, PMID: 24119307

[ref37] Pii Y. Cesco S. Mimmo T. (2015). Shoot ionome to predict the synergism and antagonism between nutrients as affected by substrate and physiological status. Plant Physiol. Biochem. 94, 48–56. doi: 10.1016/j.plaphy.2015.05.002, PMID: 26004913

[ref38] Radisky D. C. Babcock M. C. Kaplan J. (1999). The yeast frataxin homologue mediates mitochondrial iron efflux evidence for a mitochondrial iron cycle. J. Biol. Chem. 274, 4497–4499. doi: 10.1074/jbc.274.8.4497, PMID: 9988680

[ref39] Rajab H. Khan M. S. Wirtz M. Malagoli M. Qahar F. Hell R. (2020). Sulfur metabolic engineering enhances cadmium stress tolerance and root to shoot iron translocation in *Brassica napus* L. Plant Physiol. Biochem. 152, 32–43. doi: 10.1016/j.plaphy.2020.04.017, PMID: 32387912

[ref40] Robe K. Gao F. Bonillo P. Tissot N. Gaymard F. Fourcroy P.. (2020). Sulphur availability modulates Arabidopsis thaliana responses to iron deficiency. PLoS One 15:e0237998. doi: 10.1371/journal.pone.0237998, PMID: 32817691 PMC7440645

[ref41] Rogers E. E. Guerinot M. L. (2002). FRD3, a member of the multidrug and toxin efflux family, controls iron deficiency responses in Arabidopsis. Plant Cell 14, 1787–1799. doi: 10.1105/tpc.001495, PMID: 12172022 PMC151465

[ref42] Schmidt W. (2003). Iron solutions: acquisition strategies and signaling pathways in plants. Trends Plant Sci. 8, 188–193. doi: 10.1016/S1360-1385(03)00048-7, PMID: 12711231

[ref43] Shanmugam V. Wang Y. W. Tsednee M. Karunakaran K. Yeh K. C. (2015). Glutathione plays an essential role in nitric oxide-mediated iron-deficiency signaling and iron-deficiency tolerance in *Arabidopsis*. Plant J. 84, 464–477. doi: 10.1111/tpj.13011, PMID: 26333047

[ref44] Shibagaki N. Rose A. McDermott J. P. Fujiwara T. Hayashi H. Yoneyama T.. (2002). Selenate-resistant mutants of *Arabidopsis thaliana* identify Sultr1; 2, a sulfate transporter required for efficient transport of sulfate into roots. Plant J. 29, 475–486. doi: 10.1046/j.0960-7412.2001.01232.x, PMID: 11846880

[ref45] Speiser A. Silbermann M. Dong Y. Haberland S. Uslu V. V. Wang S.. (2018). Sulfur partitioning between glutathione and protein synthesis determines plant growth. Plant Physiol. 177, 927–937. doi: 10.1104/pp.18.00421, PMID: 29752309 PMC6053006

[ref46] Stemmler T. L. Lesuisse E. Pain D. Dancis A. (2010). Frataxin and mitochondrial FeS cluster biogenesis. J. Biol. Chem. 285, 26737–26743. doi: 10.1074/jbc.R110.118679, PMID: 20522547 PMC2930671

[ref47] Takahashi H. Kopriva S. Giordano M. Saito K. Hell R. (2011). Sulfur assimilation in photosynthetic organisms: molecular functions and regulations of transporters and assimilatory enzymes. Annu. Rev. Plant Biol. 62, 157–184. doi: 10.1146/annurev-arplant-042110-103921, PMID: 21370978

[ref48] Takahashi H. Watanabe-Takahashi A. Smith F. W. Blake-Kalff M. Hawkesford M. J. Saito K. (2000). The roles of three functional sulphate transporters involved in uptake and translocation of sulphate in *Arabidopsis thaliana*. Plant J. 23, 171–182. doi: 10.1046/j.1365-313x.2000.00768.x, PMID: 10929111

[ref49] Vigani G. Pii Y. Celletti S. Maver M. Mimmo T. Cesco S.. (2018). Mitochondria dysfunctions under Fe and S deficiency: is citric acid involved in the regulation of adaptive responses? Plant Physiol. Biochem. 126, 86–96. doi: 10.1016/j.plaphy.2018.02.022, PMID: 29514113

[ref50] von der Mark C. Ivanov R. Eutebach M. Maurino V. G. Bauer P. Brumbarova T. (2021). Reactive oxygen species coordinate the transcriptional responses to iron availability in *Arabidopsis*. J. Exp. Bot. 72, 2181–2195. doi: 10.1093/jxb/eraa522, PMID: 33159788 PMC7966954

[ref51] Wang H.-Y. Klatte M. Jakoby M. Bäumlein H. Weisshaar B. Bauer P. (2007). Iron deficiency-mediated stress regulation of four subgroup Ib BHLH genes in *Arabidopsis thaliana*. Planta 226, 897–908. doi: 10.1007/s00425-007-0535-x, PMID: 17516080

[ref52] Watanabe M. Hubberten H.-M. Saito K. Hoefgen R. (2010). General regulatory patterns of plant mineral nutrient depletion as revealed by serat quadruple mutants disturbed in cysteine synthesis. Mol. Plant 3, 438–466. doi: 10.1093/mp/ssq009, PMID: 20339158

[ref53] Yoshimoto N. Takahashi H. Smith F. W. Yamaya T. Saito K. (2002). Two distinct high-affinity sulfate transporters with different inducibilities mediate uptake of sulfate in *Arabidopsis* roots. Plant J. 29, 465–473. doi: 10.1046/j.0960-7412.2001.01231.x, PMID: 11846879

[ref54] Yuan Y. X. Zhang J. Wang D. W. Ling H. Q. (2005). AtbHLH29 of Arabidopsis thaliana is a functional ortholog of tomato FER involved in controlling iron acquisition in strategy I plants. Cell Res. 15, 613–621. doi: 10.1038/sj.cr.7290331, PMID: 16117851

[ref55] Zamboni A. Celletti S. Zenoni S. Astolfi S. Varanini Z. (2017). Root physiological and transcriptional response to single and combined S and Fe deficiency in durum wheat. Environ. Exp. Bot. 143, 172–184. doi: 10.1016/j.envexpbot.2017.09.002

[ref56] Zhang H. Forman H. J. (2012). Glutathione synthesis and its role in redox signaling. Semin. Cell Dev. Biol. 23, 722–728. doi: 10.1016/j.semcdb.2012.03.017, PMID: 22504020 PMC3422610

[ref57] Zuchi S. Cesco S. Varanini Z. Pinton R. Astolfi S. (2009). Sulphur deprivation limits Fe-deficiency responses in tomato plants. Planta 230, 85–94. doi: 10.1007/s00425-009-0919-1, PMID: 19350269

[ref58] Zuchi S. Watanabe M. Hubberten H.-M. Bromke M. Osorio S. Fernie A. R.. (2015). The interplay between sulfur and iron nutrition in tomato. Plant Physiol. 169, 2624–2639. doi: 10.1104/pp.15.00995, PMID: 26438787 PMC4677893

