# Supplementary figures and images for: Crosstalk Between Iron and Sulfur Homeostasis Networks in Arabidopsis
Source: Front Plant Sci. 2022 Jun 9;13:878418. doi: 10.3389/fpls.2022.878418 (PMC9224419; doi:10.3389/fpls.2022.878418)

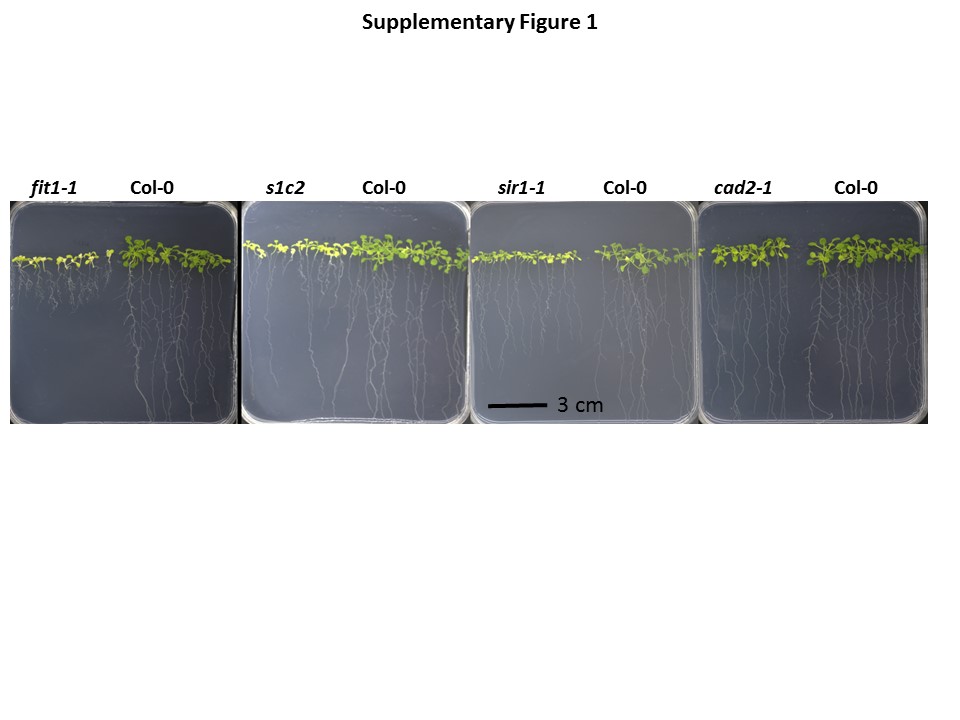

Supplement: Supplementary Figure S1 — Phenotypes of 14-days old seedlings under Fe-deficient conditions. Top view of the seedlings directly germinated on Fe-deficient half-strength MS medium for 14 days. [file Image_1.JPEG]

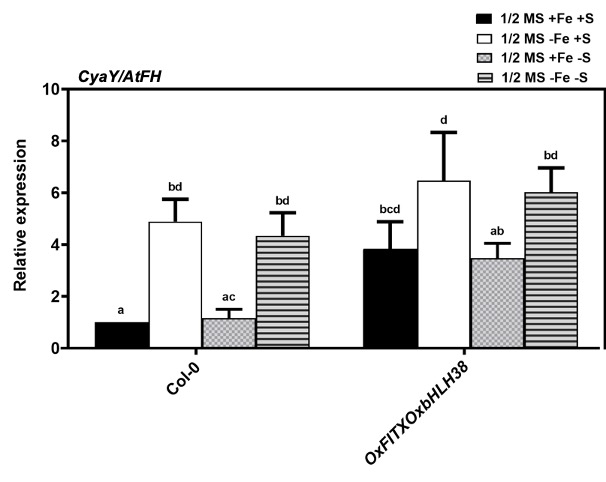

Supplement: Supplementary Figure S2 — Expression analysis of AtFH in the double overexpressor OxFIT X OxbHLH38. Relative expression by Quantitative RT-PCR of the genesCyaY/frataxin in roots,for Arabidopsis the wildtype (Col-0) and the double overexpressor line OxFITXOxbHLH38 of one-week old seedlings exposed to half-strength MS medium (1/2 MS +Fe +S), Fe deficient (1/2 MS -Fe +S), S deficient (1/2 MS +Fe -S) and Fe & S deficient (1/2 MS –Fe -S) media for four days under long-day conditions. The y-axis shows RNA levels normalized to that of GADPH. Letters indicate the statistically significant differences among all treatments in Col-0 and doubleoverxpresser line determined with the two-way ANOVA test followed by Tukey’s test (P < 0.05, n = 3). Bars represent means ± SD. [file Image_2.JPEG]
