# Supplementary material for: Crosstalk Between Iron and Sulfur Homeostasis Networks in Arabidopsis
Source: Front Plant Sci. 2022 Jun 9;13:878418. doi: 10.3389/fpls.2022.878418 (PMC9224419; doi:10.3389/fpls.2022.878418)
Supplement: Supplementary file 3 [file Table_1.docx]

Table S1: The primers used in this study

| \| **Gene symbol** \| **Sense primer 5՜--------3՜** \| **Antisense primer 5՜--------3՜** \| \| --- \| --- \| --- \| \| *Sultr1;2* \| TATCTCCGCCTTGCCTTCAC \| AGAGCGATAGTGATGGCTGC \| \| *Sultr1;1* \| CTCAAGAGCCTCGAGAAGCA \| GCGTCGCCAACAGTAAGGAA \| \| *Sultr2;1* \| GGACCAGTTGTGTTACGGGT \| TCAGTCACGACCAAGCACTC \| \| *Sultr2;2* \| CTCTTTGCCTCCGTCGAGAT \| CTCCCTAAGGCCTCAATGCT \| \| *Sultr4;1* \| CGAACTTACCGATGGAGCGA \| AAGGCCAGCTAGTTTCGCAT \| \| *Fit* \| TCCTTCTCCGGACACATACCT \| CCACAGCTTCAGGTTAGGCA \| \| *NAS1* \| TGACTCGTCTGATCTCAAAGGC \| TTAACCACGTCATCGGTTGGAT \| \| *NAS2* \| AGGAGGGGTTAGACCAATACGA \| AGCACTTCTTAGCATCACCACA \| \| *FRD3* \| GTTAGGACTTGGACTGTCCGTT \| TGAGGTGAATAACAGCAGGGTC \| \| *IRT1* \| TGGGTCTTGGCGGTTGTATC \| CCGAATGGTGTTGTTACCGC \| \| *FRO2* \| AGTACGCCACAAGAATCGCT \| CCACACTCGAACCTTCCACA \| \| *bHLH 38* \| TCCGGTTGTGGTCAAGAAGC \| TGATCTTCTTGCGTCGGTCA \| \| *CyaY/AtFH* \| ACCGTTGGATTCCTTCTGGAG \| TCCTGCAAAACCGAACTGTAA \| \| *AtSUFS/NFS2* \| TTGCTCCTCTTCGCTATCCG \| ACCCATTCACTTCCTGGTGG \| \| *AtNifS1/IscS* \| ACCAGGGTTTCAGAGACGAC \| CGTGAAGAAGAAGAAGATGCGAG \| \| *GADPH*  *AtFH-ChIP-a*  *AtFH-ChIP-b*  *AtFH-ChIP-c*  *AtFH-ChIP-d*  *AtFH-ChIP-e*  *AtACT8-ChIP* \| GTTGATCTCACCGTTAGACTTGAG  AAGCAATCAAATCCGACTGG  CACCATCAGCATTTCCCATA  GCCCTTCATTTCTTCAGTCG  CACCTGAGTGCATAGCGAAA  CCGAGGTTTCTCAAGCTGTC  CGACCCTATCACAGTCAATCC \| CGTTGTCGTACCATGACACCAACT  ACCTCTGGGGGTTGAATTTG  ATCGGTCAAGCGTTGTTCTT  TATGCAAGTTATGGCGCTTG  TACCCGGTTCACGTATTTCC  TCTCCAGAAGGAATCCAACG  AACGTGACATGGCTGTCAGA \| |  |  |
| --- | --- | --- | --- | --- | --- | --- | --- | --- | --- | --- | --- | --- | --- | --- | --- | --- | --- | --- | --- | --- | --- | --- | --- | --- | --- | --- | --- | --- | --- | --- | --- | --- | --- | --- | --- | --- | --- | --- | --- | --- | --- | --- | --- | --- | --- | --- | --- | --- | --- | --- | --- | --- | --- |
